# Supplementary material for: Divide-and-conquer: machine-learning integrates mammalian and viral traits with network features to predict virus-mammal associations
Source: Nat Commun. 2021 Jun 25;12:3954. doi: 10.1038/s41467-021-24085-w (PMC8233343; doi:10.1038/s41467-021-24085-w)
Supplement: Supplementary file 3 — Reporting Summary [file 41467_2021_24085_MOESM3_ESM.pdf]

nature research

Corresponding author(s): Maya Wardeh

Last updated by author(s): May 10, 2021

Reporting Summary

Nature Research wishes to improve the reproducibility of the work that we publish. This form provides structure for consistency and transparency in reporting. For further information on Nature Research policies, see our Editorial Policies and the Editorial Policy Checklist.

Statistics

For all statistical analyses, confirm that the following items are present in the figure legend, table legend, main text, or Methods section.

n/a	Confirmed	
☐	☒	The exact sample size (n) for each experimental group/condition, given as a discrete number and unit of measurement
☐	☒	A statement on whether measurements were taken from distinct samples or whether the same sample was measured repeatedly
☒	☐	The statistical test(s) used AND whether they are one- or two-sided<br>*Only common tests should be described solely by name; describe more complex techniques in the Methods section.*
☐	☒	A description of all covariates tested
☐	☒	A description of any assumptions or corrections, such as tests of normality and adjustment for multiple comparisons
☐	☒	A full description of the statistical parameters including central tendency (e.g. means) or other basic estimates (e.g. regression coefficient) AND variation (e.g. standard deviation) or associated estimates of uncertainty (e.g. confidence intervals)
☒	☐	For null hypothesis testing, the test statistic (e.g. F, t, r) with confidence intervals, effect sizes, degrees of freedom and P value noted<br>*Give P values as exact values whenever suitable.*
☒	☐	For Bayesian analysis, information on the choice of priors and Markov chain Monte Carlo settings
☐	☒	For hierarchical and complex designs, identification of the appropriate level for tests and full reporting of outcomes
☐	☒	Estimates of effect sizes (e.g. Cohen's d, Pearson's r), indicating how they were calculated

Our web collection on statistics for biologists contains articles on many of the points above.

Software and code

Policy information about availability of computer code

Data collection	No specialised software was used in data collection.
Data analysis	R version 3.6.2 was used for data analysis. All models used (GBM, random forest (ranger), AvNNEt, and support vector machines) were trained and optimised using caret R package (version 6.0.85). All codes used in our analyses are made available via figshare (https://doi.org/10.6084/m9.figshare.13270304).

For manuscripts utilizing custom algorithms or software that are central to the research but not yet described in published literature, software must be made available to editors and reviewers. We strongly encourage code deposition in a community repository (e.g. GitHub). See the Nature Research guidelines for submitting code & software for further information.

Data

Policy information about availability of data

All manuscripts must include a data availability statement. This statement should provide the following information, where applicable:

- Accession codes, unique identifiers, or web links for publicly available datasets
- A list of figures that have associated raw data
- A description of any restrictions on data availability

Virus-mammal species-level associations were obtained from the ENHanCEd Infectious Diseases Database (EID2: https://eid2.liverpool.ac.uk/). Virus taxonomy was obtained from the NCBI Taxonomy database (https://www.ncbi.nlm.nih.gov/Taxonomy). Viral traits were assembled from the NCBI Taxonomy database, the International Committee on Taxonomy of Viruses ICTV (https://talk.ictvonline.org/) and ViralZone (https://viralzone.expasy.org/). Mammalian and geospatial data were obtained from open-access data sources. These sources are listed in detail, and their DOIs provided in the Supplementary Information file. Data used can be found here: https://doi.org/10.6084/m9.figshare.13270304, except for mammalian presence shapefiles and raw climate data (due to their large size) - these data can be obtained from the authors or directly from the sources listed in the Supplementary Information file.

Field-specific reporting

Please select the one below that is the best fit for your research. If you are not sure, read the appropriate sections before making your selection.

☐ Life sciences ☐ Behavioural & social sciences ☒ Ecological, evolutionary & environmental sciences

For a reference copy of the document with all sections, see nature.com/documents/nr-reporting-summary-flat.pdf

Ecological, evolutionary & environmental sciences study design

All studies must disclose on these points even when the disclosure is negative.

Study description

Our knowledge of viral host ranges remains limited. Completing this picture by identifying unknown hosts of known viruses is an important research aim that can help identify and mitigate zoonotic and animal-disease risks, such as spill-over from animal reservoirs into human populations. To address this knowledge-gap we apply a divide-and-conquer approach which separates viral, mammalian and network features into three unique perspectives, each predicting associations independently to enhance predictive power. A suite of machine-learning models are trained in each perspective, and their results are aggregated via majority voting to generate final predictions.

Research sample

Documented associations between viruses and their mammalian hosts were obtained from the Enhanced Infectious Diseases Database (EID2: https://eid2.liverpool.ac.uk/)(Wardeh et al, 2015). EID2 is the largest source of pathogen-host association, and is fully evidence based (PubMed publications and/or meta-data associated with genomes uploaded to NCBI Nucleotide database).

Sampling strategy

We used as many mammalian and viral species (found in mammals) as there were data for. viruses found in other host species (e.g. avian hosts, invertebrates) were excluded from this study.

Data collection

Virus-mammal associations were downloaded from the ENHanCEd Infectious Diseases Database (EID2: https://eid2.liverpool.ac.uk/ - December/2019). These associations were aggregated to species level so that if a virus strain were found in a mammalian subspecies, the species level association between the corresponding virus and the mammalian species were included in the study. Virus species hierarchies were obtained from the NCBI Taxonomy database (December/2019); mammalian taxonomy was obtained from EID2. Viral traits were assembled from the NCBI Taxonomy database, the International Committee on Taxonomy of Viruses ICTV (https://talk.ictvonline.org/) and ViralZone (https://viralzone.expasy.org/). Mammalian traits and geospatial data were obtained from open-access data sources as listed in detail in Supplementary Notes 1-3 of the Supplementary Information file.
Mammalian and viral data were disambiguated to match species names used in the study.

Timing and spatial scale

Virus-mammal species associations were obtained in single (one-off) download from EID2, version from Dec 2019. Viral traits were obtained for included viruses within the same time-frame.Timing scale for mammalian traits is as per their original sources. Original resolutions of geospatial data are listed in Supplementary Table 3; these data were extrapolated to 5 km squared prior to calculating geospatial features.

Data exclusions

We excluded non-terrestrial mammals from our analyses due to non-availability of geo-spatial traits (such as land-cover type, human population). These exclusion criteria were pre-established.

Reproducibility

Results are fully reproducible by running the codes are made available via figshare (https://doi.org/10.6084/m9.figshare.13270304). All included experiments are computational. Our pipelines included 50 replicate of each mammalian and viral model, and 100 replicate of each network-based model.

Randomization

No randomisation was applied during data collection. Models' inputs were randomly sampled as per the sampling routines discussed in the methods section. Test sets were randomly sampled (15% of all associations).

Blinding

Blinding was not relevant for our study, we used all relevant data (with the exception of non-terrestrial mammals, which were removed prior to analyses).

Did the study involve field work? ☐ Yes ☒ No

Reporting for specific materials, systems and methods

We require information from authors about some types of materials, experimental systems and methods used in many studies. Here, indicate whether each material, system or method listed is relevant to your study. If you are not sure if a list item applies to your research, read the appropriate section before selecting a response.

Materials & experimental systems

n/a	Involved in the study
☒	☐ Antibodies
☒	☐ Eukaryotic cell lines
☒	☐ Palaeontology and archaeology
☒	☐ Animals and other organisms
☒	☐ Human research participants
☒	☐ Clinical data
☒	☐ Dual use research of concern

Methods

n/a	Involved in the study
☒	☐ ChIP-seq
☒	☐ Flow cytometry
☒	☐ MRI-based neuroimaging
