# Supplementary material for: Divide-and-conquer: machine-learning integrates mammalian and viral traits with network features to predict virus-mammal associations
Source: Nat Commun. 2021 Jun 25;12:3954. doi: 10.1038/s41467-021-24085-w (PMC8233343; doi:10.1038/s41467-021-24085-w)
Supplement: Supplementary file 4 — Description of Additional Supplementary Files [file 41467_2021_24085_MOESM4_ESM.pdf]

Description of Additional Supplementary Files

File Name: Supplementary Data 1
Description: **Top 60 predicted or detected wild or semi-domesticated hosts of West Nile Virus (WNV).** Host species were ordered by mean probability of predictions derived as probability from mammalian, viral and network perspectives, and top 60 were selected. Dom is domestication status, w=wild, sd = semi domestic. EID2 indicates if the association was retrieved from EID2 (=1). Predicted indicates if association was predicted (via voting using median probability ≥0.5 =1 from three perspective). Mammalian lists median probabilities drawn from mammalian perspective (50 replicate models per host with two or more known viruses (n=699) trained with viral traits). Viral lists median probabilities drawn for WNV from viral perspective (50 replicate WNV models trained with mammalian traits). Network lists median probabilities drawn from network perspective (100 replicate models trained with motif features). Mean lists mean probability across the three perspective.

File Name: Supplementary Data 2
Description: – **Top 50 detected (in EID2) or predicted virus species that could be found in *Rousettus leschenaultii.*** Virus species are ordered by mean probability of predictions derived as probability from mammalian, viral and network perspectives, and top 50 were selected. Abb indicates the abbreviation of the virus (if any). Baltimore is Baltimore classification (7 groups I to VII). EID2 indicates if the association was retrieved from EID2 (=1). Predicted indicates if the association was predicted (via voting using median probability >0.5 =1 from three perspective). Viral lists median probabilities drawn for *R. leschenaultii* from viral perspective (50 replicate models per virus (n=556) trained with mammalian traits). Mammalian lists median probabilities drawn from mammalian perspective (50 replicate models for *R. leschenaultii* trained with viral traits). Network lists median probabilities drawn from network perspective (100 replicate models trained with motif features). Mean lists mean probability across the three perspective.

File Name: Supplementary Data 3
Description: – **Details of viruses in Figure2-B and Supplementary Figure 8-B.** Virus species were ordered (in each figure) in descending order of their predicted host range and top 40 were selected. Rabies lyssavirus was excluded from the figures and is included here for completion. Values in brackets represent 90% confidence intervals. Columns with research effort indicate results obtained with research effort incorporated into viruses and mammals included in viral and mammalian perspective models, respectively.

File Name: Supplementary Data 4
Description: **Predictions for mammalian orders.** Rabies virus was excluded from the counts of observed, predicted associations, and association fold-increase. Zoonotic refers to associations with viruses known to affect humans. Domestic refers to associations with viruses known to affect economically important domesticated mammals (livestock - including camels, horses, and pets - including rabbits). Columns with research effort indicate results obtained with research effort incorporated into viruses and mammals included in viral and mammalian perspective models, respectively.

File Name: Supplementary Data 5
Description: **Details of mammalian species in Figure 3-B and Supplementary Figure 9-B.** In each figure, labelled mammals are as follows: top 4 (by number of new viruses) for each of Artiodactyla, Carnivora, Chiroptera, Primates, Rodentia, and other orders. Values in brackets represent 90% confidence intervals. Columns with research effort indicate results obtained with research effort incorporated into viruses and mammals included in viral and mammalian perspective models, respectively.

File Name: Supplementary Data 6
Description: **Predictions for virus genus and mammalian order associations.** Rabies virus was excluded from the counts of observed, predicted associations, and association fold-increase. Zoonotic refers to associations with viruses known to affect humans. Domestic refers to associations with viruses known to affect economically important domesticated mammals (livestock - including camels, horses, and pets - including rabbits). Values in brackets represent 90% confidence intervals. Columns with research effort indicate results obtained with research effort incorporated into viruses and mammals included in viral and mammalian perspective models, respectively.
